# Diseases of the Abdomen

**Published:** 1902-11-29

**Authors:** 


### Diseases of the Abdomen.

(*Concluded from page* 115.)

Kruse's bacillus of dysentery[12] has been found in further cases at Laar, Barmen, in an asylum at Dusseldorf, and at Utrecht. The bacilli are agglutinated by the serum of dysenteric convalescents in a dilution of 50 to 200, the control tests being negative. An assistant in the laboratory working at this bacillus developed a typical attack, though he had not come into contact with a natural means of infection. Flexner's and Shiga's bacilli are probably varieties. He considers asylum dysentery a different disease from the acute tropical form. To combat epidemics isolation and disinfection are necessary, though the importance of water as a means of conveying infection is not so important.

In the treatment of asylum dysentery Macdonald[13] has tried with success very weak solutions of permanganate of potash used twice a day with an injection syringe. The strength used was gr. ij.–iv. to the pint. The cases all rapidly improved, and apparently the action is a double one, a styptic and disinfecting agent.

In the recent epidemic of dysentery in Grenada Duprey[14] has been using a treatment in which sulphur and Dover's powder have largely figured with marked success. The result of giving a powder composed as follows:

Sub. sulphuris, gr. 20
Pulv. ipecac. co., gr. 5 to 10

every four hours was that after six powders blood and mucus ceased from the motions, and straining was much lessened, the motions being less frequent and more fluid. Griping pains had to be combated by morphia. Sips of very cold water and occasionally a mustard poultice to the epigastrium were of service. Ipecacuhana, he says, is difficult to administer, but where it could be used fairly it gave good results. Salines used early were tried, but in his hands the sulphur method gave by far the best results.

Hale White and Golding Bird[15] give us the subsequent history of three cases in which right lumbar colotomy has been done for colitis. Two of the patients remained perfectly cured, but the third relapsed after 18 months. They urge that the colotomy wound must be left open a long time. In these cases there were periods of 1, 1½, and 2½ years since operation. Severe cases might require as long as 10 years. The right lumbar operation is better than cæcotomy. Irrigation is harmful in membranous colitis, beneficial in the ulcerative form. While he was medical officer to the hospital in Jerusalem, London[16] came across a number of cases of myiasis intestinalis, with dysenteric symptoms of extreme obstinacy. Calomel and santonin removed the larvæ when the case was diagnosed. He has noticed that the infection is strongest at the time that the water-pools are at their lowest; this time is also that of the swarming of the diptera. He thinks that there is here a relation of cause and effect between the insects and the disease through the water.

In Grenada cases of coma have been observed by Duprey[17] which were unassociated with albuminuria, malaria, or alcoholism, but were relieved immediately by remedying the condition of constipation that such patients were victims to. They seemed to be cases of auto-intoxication. He describes the case of a child, aet. 6, in whom the only abnormal condition found post mortem was that of extreme constipation, and he supposes that the toxicity in this case was so extreme that death was caused without intervening coma.

By a system of careful dilution Gordon[18] has been able to estimate in round numbers the bacteria in fæces. After the administration of izal oil by the mouth the total number of micro-organisms present in a gramme of fæces and developing in agar-plates at blood heat was reduced by

86 per cent. in Exp. I.
90 per cent. in Exp. II.
60 per cent. in Exp. III.

The pathogenicity of No. 1 was reduced also 90 per cent. This was only to be attributed to the action of the izal oil, and the result was broadly proportionate to the dose. He believes that the use of izal is justified as a powerful intestinal antiseptic in doses that give rise to no untoward symptoms. The fact that even in spite of great reduction the number of micro-organisms remains still large leads him to ask whether possibly a relatively small disturbance of equilibrium in the bacteria of the intestine may not lead to a marked change in the state of health. The indication would be then to re-establish this equilibrium as rapidly as possible, and a drug which will accomplish this without injury, pain, or inconvenience is of great value. Its value is unquestioned in diarrhœa and dysentery ; it is possible that enteric fever and appendicitis may be in some way controlled by such intestinal antiseptics. Izal is more efficacious than β-naphthol or salol.

[12] Brit. Med. Jour., March 1. [13] Lancet, March 1. [14] Jour. of Tropical Medicine, July 1. [15] Brit. Med. Jour., May 31. [16] Brit. Med. Jour., March 15. [17] Lancet, June 28. [18] Lancet, March 8.

### PROGRESS IN PHOTOTHERAPY.

(*Concluded from page* 130.)

